# Developing and validating the Psychosocial Burden among people Seeking Abortion Scale (PB-SAS)

**DOI:** 10.1371/journal.pone.0242463

**Published:** 2020-12-10

**Authors:** M. Antonia Biggs, Torsten B. Neilands, Shelly Kaller, Erin Wingo, Lauren J. Ralph

**Affiliations:** 1 Advancing New Standards in Reproductive Health, Bixby Center for Global Reproductive Health, Department of Obstetrics, Gynecology and Reproductive Sciences, University of California San Francisco, San Francisco, California, United States of America; 2 Division of Prevention Science, Department of Medicine, University of California San Francisco, San Francisco, California, United States of America; Institute of Mental Health, SINGAPORE

## Abstract

While there is a large body of research demonstrating that having an abortion is not associated with adverse mental health outcomes, less research has examined which factors may contribute to elevated levels of mental health symptoms at the time of abortion seeking. This study aims to develop and validate a new tool to measure dimensions of psychosocial burden experienced by people seeking abortion in the United States. To develop scale items, we reviewed the literature including existing measures of stress and anxiety and conducted interviews with experts in abortion care and with patients seeking abortion. Thirty-five items were administered to 784 people seeking abortion at four facilities located in three U.S. states. We used exploratory factor analysis (EFA) to reduce items and identify key domains of psychosocial burden. We assessed the predictive validity of the overall scale and each sub-scale, by assessing their associations with validated measures of perceived stress, anxiety, and depression using multivariable linear regression models. Factor analyses revealed a 12-item factor solution measuring psychosocial burden seeking abortion, with four subdomains: structural challenges, pregnancy decision-making, lack of autonomy, and others’ reactions to the pregnancy. The alpha reliability coefficients were acceptable for the overall scale (α = 0.83) and each subscale (ranging from α = 0.82–0.85). In adjusted analyses, the overall scale was significantly associated with stress, anxiety and depression; each subscale was also significantly associated with each mental health outcome. This new scale offers a practical tool for providers and researchers to empirically document the factors associated with people’s psychological well-being at the time of seeking an abortion. Findings suggest that the same restrictions that claim to protect people from mental health harm may be increasing people’s psychosocial burden and contributing to adverse psychological outcomes at the time of seeking abortion.

## Introduction

A large body of evidence from the U.S. and internationally, has firmly demonstrated that abortion does not increase people’s mental health risk when compared to giving birth [1–5]. However, these studies also suggest that mental health symptoms are higher around the time of seeking an abortion—whether or not they obtained one [1,2]. The U.S. Turnaway study interviewed nearly 1,000 people seeking abortion and compared the mental health outcomes of those who obtained an abortion to people who were denied one, and followed them for five years. While having an abortion did not increase people’s risk of experiencing psychological symptoms at any point during the five-year study period, mental health symptoms were higher around the time of seeking an abortion, when compared to years later [1]. The source of people’s higher symptom levels at the time of seeking an abortion when remains unanswered.

There are a number of personal and external factors—including the reasons for seeking abortion and perceived abortion stigma—that may increase psychological symptoms at the time of seeking an abortion when compared to other moments in people’s lives. People’s reasons for seeking abortion are often driven by their concerns for current and future children, family, as well as existing commitments and responsibilities [6,7]. Being confronted with an unintended pregnancy in the face of competing demands, concerns about the well-being of loved ones, lack of social support, and/or lack of financial or emotional resources to obtain pregnancy-related care or to parent a new baby may contribute to feelings of distress. Furthermore, people considering abortion may experience abortion stigma which is associated with negative post-abortion emotions and mental health symptoms [8,9].

Furthermore, the added burden of state-level policies such as mandated waiting period and counseling laws, which claim to protect people from mental health harm, have the potential to increase levels of stress and anxiety [10]. Currently, 27 states have mandated waiting period laws, 18 states have mandated counseling laws, and eight states require people seeking abortion to be warned of the negative psychological and emotional responses to abortion [10]. In Texas, people are told that they may experience recurring depression, thoughts of suicide, and anxiety after an abortion, despite the large body of evidence demonstrating the contrary [11–15]. These laws can lead to delays or prevent people from obtaining abortion care and do not consider whether exacerbating the burdens that people experience seeking abortion care may in of themselves cause mental health harm [16–19].

The research examining the association between barriers accessing abortion care and mental health remains limited. Interviews with people seeking abortion in Michigan and New Mexico found that many people experienced delays accessing care due to travel, financial, and other logistical barriers which they believed had negative implications for their mental health [19]. In Texas, minors seeking judicial bypass in order to avoid the state’s parental consent requirement described the experience as “intimidating” and “scary” [20]. While findings from the Turnaway study showed that being denied an abortion was associated with short-term elevated levels of stress and anxiety, when compared to obtaining an abortion, the specific source of symptoms was not explored [1,3]. This study aims to develop and validate a unique measure of psychosocial burden among people seeking abortion in the United States that will help to assess and identify the array of factors that comprise the psychosocial burden experienced by people seeking abortion.

## Materials and methods

### Item generation

We defined *psychosocial burden* as a person’s *subjective* perception of the burden experienced seeking abortion care, including the perceived difficulty overcoming logistical barriers to care, as well as worry about a range of socio-emotional factors that may have a negative impact on their psychological well-being. Our concept of burden is drawn from Lazarus’ stress theory of cognitive appraisal [21], where the physiological response of stress occurs if a situation is appraised as negative. Our measure was designed to assess whether structural barriers experienced trying to obtain abortion care, as well as individual-level factors, such as social support and pregnancy decision-making, are appraised as negative. In developing our items, we used both an inductive and deductive approach, as described by Hinkin, Tracey, and Enz [22]. For deductively developed items, we drew from validated measures of stress and anxiety including the Daily Hassles Scale, a widely used measure of stress that has been shown to be a reliable predictor of current and future psychological symptoms, as well as measures of pregnancy-related stress [23–26] and adapted them to the abortion context. Additionally, we ensured that each item addressed only one issue (i.e., the item was not double barreled), and was brief and clear. To limit cognitive burden in completing items, we avoided reverse-scored items and included some overlap among items. We developed an initial list of 55 items that related to timing, financial, travel and other access-related challenges as well as social support and decision-making related to the pregnancy and abortion.

We invited 11 experts who work closely with or study people seeking abortion to review and add to our list of items. Experts included four clinicians who provide abortion care, two abortion counselors, one clinic director, three abortion researchers, and one lawyer who supports youth in navigating the judicial bypass process. We met with each expert individually to discuss items, solicit suggestions for rewording and to identify new items and domains.

After expert review, we revised existing items, added new topic areas, and removed items that were viewed as redundant or too sensitive, resulting in a revised set of items. We further tested these 45 items through cognitive interviews with 11 patients seeking abortion at three abortion facilities located in the San Francisco Bay Area. The cognitive interviews began with the open-ended question “In thinking about the many challenges people face accessing abortion care, what do you think are some of the most difficult aspects of seeking abortion care?” We then presented cognitive interview participants with a list of 45 structured four-point Likert-scaled items, regarding how difficult, worried, or relieved they felt about a range of topics since they discovered they were pregnant. Each item also included an “other” category for the participant to write in a response. To test understanding of each item, we asked participants to describe the items in their own words and whether any items were confusing or difficult to understand. We revised these items iteratively, as we conducted the cognitive interviews. We tested a final set of 35 items on a larger population of people seeking abortion care. Using the criteria set forth by Nunnally and Bernstein [27] to include at least 10 people per item in the sample, we estimated that a sample of at least 350 would be sufficient to test our 35 items.

### Recruitment procedures

From January to June 2019, we approached 1092 potential participants, 846 agreed to participate, 20 were ineligible, 824 of those eligible initiated the survey, and a total of 784 completed at least one-fifth of the survey, allowing for sufficient responses for analyses. Although most people did not give a reason for not participating (n = 246), the most common reasons included not interested (n = 33), not comfortable or stressed (n = 28), and not a good time (n = 27). We recruited study participants from four abortion facilities located in three states (California, Illinois, and New Mexico) that had public funding for abortion services, no mandated counseling or waiting period laws, and provided abortions beyond the first trimester. These recruitment sites were selected because we aimed to oversample people who may have traveled across state lines to avoid the abortion restrictions in the state where they live. To be eligible, people had to be seeking an abortion at the time of recruitment, aged 15 years or older, and able to speak and read English or Spanish. People who had already completed their abortion or were pre-medicated with narcotics prior to the abortion procedure were considered ineligible. Clinic staff notified research staff if a patient was pre-medicated so that those patients were not approached for study participation. Research staff introduced the study to patients while they were waiting for their abortion appointment, handed interested patients a tablet device to complete and confirm their eligibility, and consented those eligible and interested to participate in the study. Participants self-administered an anonymous survey programmed in Qualtrics, which they could choose to complete in either English or Spanish, with research staff available to assist as needed. Participants received a $30 gift card as remuneration. This study received ethical approval from the University of California, San Francisco’s Institutional Review Board.

### Scale development

To establish preliminary construct validity, we used exploratory factor analysis (EFA) using the iterated principal-factor method, with oblique rotation. EFA allowed us to reduce the number of scale items and to identify underlying scale dimensions. In our EFA analysis, we used multiple imputation methods in Stata 15 to address missing data, which ranged from 3 (0.38%) to 15 (1.9%) missing cases, depending on the item. We extracted the number of factors by visualizing where eigenvalues began to level off after a significant drop using a scree plot [28]. After rotating factors, we removed items with a factor loading less than 0.40 and uniqueness greater than 0.60 [29]. The item’s uniqueness refers to the percentage of variance for the item that is not explained by the common factors. After removing items based on these criteria, we re-ran the factor analysis and assessed the usefulness and interpretability of each factor until we arrived at a final factor structure solution (Table 2). Following factor analyses, we conducted a Kaiser-Meyer-Olkin test to assess whether the variables have enough in common to warrant factor analysis. We used a post-estimation command to produce the correlations of common factors and used pairwise correlations to assess the associations between the full scale and each factor. To score the full scale and each sub-scale, we calculated mean scores across items for people who had responded to at least half of the full scale or sub-scale, with higher scores indicating greater psychosocial burden. We considered internal consistency alpha reliability scores above .70 acceptable [30].

To evaluate whether the resulting scale should be treated as a series of separate sub-scales versus a single overall scale, we performed two confirmatory factor analyses (CFAs) using Stata’s sem command. The first CFA reprised the EFAs’ factor structure with multiple correlated lower-order factors. The second CFA fitted a higher-order CFA in which the lower-order factors were treated as indicators of a single higher-order general factor. The goodness of fit of each CFA were evaluated using the Comparative Fit Index (CFI) and the Root Mean Square Error of Approximation (RMSEA) [31,32]. Following Hu and Bentler (1999), we treated CFI ≥ .95 and RMSEA ≤ .06 as indicative of satisfactory model-data fit [32,33]. We compared the two CFAs using the Bayesian Information Criterion (BIC), using Raftery’s criteria of values of 2 or lower indicating a negligible difference, 2–5 signifying a positive difference, 5–10 indicating a strong difference, and a difference in excess of 10 indicating a very strong difference [34].

#### Scale validation

We assessed the predictive validity of the scale items through multivariable linear regression analyses with multiple imputation then deletion methods, using chained equations [35]. In adjusted analyses, we assessed whether the overall scale and each sub-scale was associated with validated measures of perceived stress, anxiety, and depression. These included Cohen’s Perceived Stress Scale [25], a measure based on the sum of four Likert-scaled items (ranging from never to very often) with scores ranging from 0 to 16 (α = .62). Anxiety symptoms were measured using the Generalized anxiety disorder (GAD-7) scale, which consists of seven Likert-scaled items, with total scores ranging from 0 to 21 (α = .94), and scores of 15 and over considered at risk of an anxiety disorder [36]. Depressive symptoms were measured using the Patient Health Questionnaire-2 (PHQ2), a measure based on the sum of two Likert-scaled items assessing symptoms over the last two weeks (α = .86). PHQ2 scores can range from 0 to 6, with scores of 3 or greater considered at risk of a major depressive disorder [37]. Moderate, yet significant associations between the scale and these mental health measures indicate that the full scale is related but distinctive from validated measures of stress, anxiety and depression.

Our final scale and each of its sub-scales served as our primary independent variables of interest. Model covariates included demographic characteristics such as age group (categorical), race/ethnicity (categorical), receipt of any government assistance (Temporary Assistance to Needy Families, WIC, food stamps, social security/disability or other) in the past year (dichotomous), and confidence participants could come up with $2,000 if an unexpected need arose within month (not at all confident, only slightly confident, somewhat confident, very confident). We also controlled for a priori factors known to be associated with adverse mental health outcomes (history of depression or anxiety, and any adverse childhood experiences, as measured by a selection of 5 items on the Adverse Childhood Experiences Questionnaire (ACE-Q) [38]). ACEs asked whether participants at age 17 or younger lived with anyone who was severely depressed, mentally ill, had a drinking or drug problem or served time in jail, whether they witnessed violence in their neighborhood, and whether they often felt unsupported, unloved, or unprotected at home [38]. We also examined current and previous pregnancy characteristics including how they felt about becoming pregnant before they became pregnant (wanted pregnancy later, sooner, wanted pregnancy then, didn’t want to be pregnant then or at any time in the future, and not sure), gestational age at the time of recruitment according to self-reported date of their last menstrual period (LMP) or number of weeks pregnant if LMP date unknown, reason for seeking abortion (fetal anomaly, rape, or other reasons), parity, and relationship with the man involved in the pregnancy (very committed vs somewhat committed/not in an intimate relationship). Education was not included as a model covariate because it was highly correlated with age and thus not independent.

We ran additional multivariable analyses with and without the full scale, to assess the relative contribution of the full scale on predicting each mental health outcome. We estimated effect sizes or strength of the relationship between the overall scale and subscales and mental health using Cohen’s f^2^ where f^2^ ≥ 0.01, f^2^ ≥ 0.06, and f^2^ ≥ 0.16 represent small, medium, and large effect sizes, respectively [39]. We repeated these same analyses for each sub-scale.

## Results

Participant characteristics are presented in Table 1. Most participants were in their twenties (54%), over one-fourth identified as non-Hispanic white (27%) and non-Hispanic black (27%).

**Table 1:** Participant characteristics (N = 784).

**Demographics**	**%**	**N**
Age group		
*15–19*	*12*	*97*
*20–24*	*25*	*199*
*25–29*	*29*	*230*
*30–34*	*18*	*140*
*35–45*	*15*	*117*
*Missing*	<1	1
**Race/ethnicity**		
*White (non-Hispanic)*	27	208
*Black*	27	208
*Hispanic/Latina*	23	179
*Asian/Pacific Islander*	6	46
*Other /Mixed race*	11	86
*Missing*	7	57
Marital status		
*Never married*	72	562
*Married*	11	85
*Separated/Divorced/Widowed*	9	72
*Missing*	8	65
Highest level of education		
*Less than high school*	11	83
*High school or equivalent*	28	221
*Some college/Associates/Technical school*	37	288
*College degree or higher*	17	132
*Missing*	8	60
**Current pregnancy and pregnancy history**		
Retrospective pregnancy intentions		
*Mistimed (wanted pregnancy sooner/later)*	34	270
*Pregnancy wanted*	4	33
*Wanted pregnancy never*	42	326
*Not sure what wanted*	19	152
*Missing*	<1	3
Lives more than 25 miles from recruitment clinic	388	49
Gestational age at time of recruitment (last menstrual period)		
*< = 12 wks*.	70	548
*13–19 wks*.	14	112
*> = 20 wks*.	14	113
*Missing*	1	11
Seeking abortion because pregnancy result of rape	2	14
Seeking abortion because fetus has medical condition	4	30
Parity		
*Nulliparous*	37	292
*Parous*	55	432
*Missing*	8	60
In a very committed intimate relationship with man involved in pregnancy	48	373
**Socioeconomic status**	**%**	**N**
How confident could come up with $2,000 if an unexpected need arose within month		
*Not at all confident*	45	356
*Only slightly confident*	20	155
*Somewhat confident*	15	121
*Very confident*	10	79
*Missing*	9	73
Household received any government assistance, last year		
*No*	50	395
*Yes*	42	331
*Missing*	7	58
**Adverse childhood events and mental health history**		
Ever diagnosed with anxiety or depression	25	196
*Missing*	8	63
Had 4 or more drinks on one occasion, at least monthly, past year (pre-pregnancy)	30	233
*Missing*	8	61
Used illicit or street drugs on one occasion, at least monthly, past year (pre-pregnancy)	13	99
*Missing*	8	65
History of adverse childhood experiences		
*One or more adverse childhood experiences*	34	265
*None*	60	470
*Missing*	6	49
**Clinic where patient accessed services**		
*A*	32	248
*B*	27	214
*C*	27	212
*D*	14	110

The factor analyses revealed a final 12-item, four factor solution (Table 2). Result of the Kaiser-Meyer-Olkin test was 0.80, suggesting good sampling adequacy and that our data were well-suited for factor analysis. The alpha reliability coefficients for the overall scale (α = 0.83) and each sub-scale (ranging from α = 0.82–0.85) were acceptable and could not be improved by dropping additional items. We labeled our overall scale “Psychosocial Burden among people Seeking Abortion Scale (PB-SAS)” and called our first factor “Structural challenges” because it includes items related to the difficulty finding and traveling to a place to obtain abortion care (see S1 Table for scale items). The second factor “Pregnancy decision-making” includes the difficulty thinking you have to have an abortion, deciding to have an abortion and worry about ending a potential life. The third factor “Lack of autonomy” includes three items related to feeling forced to tell people about the pregnancy, abortion, and having to wait to have the abortion. Factor four, “Others’ reactions to the pregnancy” includes two items related to worry about parents, friends or other family members’ reactions to the pregnancy (Table 2). The mean scores for the full scale were 0.90, 0.81 for Structural Challenges, 1.44 for Pregnancy Decision-Making, 0.32 for Lack of Autonomy, and 1.8 for Others’ Reactions. The proportion of variance explained by each factor ranged from 29% for Factor 4 to 39% for Factor 1 (Table 2). It is important to note that with oblique rotation the variances explained by each factor intersect and should not be summed.

**Table 2:** Exploratory factor analysis (EFA) using the iterated principal factor method, factor loadings, means and standard deviations (SD) (n = 774).

Factors and items	Factor loading	Mean (SD) (range 0–3)
**Factor 1. Structural challenges** *(not at all difficult—very difficult)*		0.81(0.84)
Finding a place to obtain care to end this pregnancy	0.82	0.77(1.01)
Traveling to a place to obtain care to end this pregnancy	0.80	0.87(1.07)
Amount of time I have spent trying to obtain care to end this pregnancy	0.73	1.01(1.08)
Scheduling an appointment to end this pregnancy	0.72	0.59(0.91)
Cronbach’s α = 0.85, eigenvalue 3.99, 39% explained variance		
**Factor 2. Pregnancy decision-making**		1.44(1.08)
Thinking I have to end this pregnancy *(not at all difficult—very difficult)*	0.93	1.44(1.21)
Deciding whether to end this pregnancy *(not at all difficult—very difficult)*	0.93	1.47(1.20)
I’m ending a potential life *(not at all worried—very much worried)*	0.57	1.39(1.25)
Cronbach’s α = 0.85, eigenvalue 1.56, 34% explained variance		
**Factor 3. Lack of autonomy** *(not at all—very much)*		0.32(0.63)
Felt forced to tell people that I was considering ending this pregnancy	0.89	0.32(0.74)
Felt forced to tell people that I was pregnant	0.82	0.28(0.70)
Felt forced to wait to end this pregnancy after I had made a decision	0.61	0.36(0.77)
Cronbach’s α = 0.82, eigenvalue 1.27, 34% explained variance		
**Factor 4. Others’ reactions to the pregnancy** *(not at all worried—very much worried)*		1.18(1.15)
My parents’ or guardians’ reaction to the pregnancy	0.88	1.26(1.27)
My friends’ or other family members’ reaction to the pregnancy	0.86	1.09(1.18)
Cronbach’s α = 0.85, eigenvalue 1.00, 29% explained variance		
**Full scale**. 12 Items, overall Cronbach’s α = 0.83		0.90(0.62)

SD = Standard deviation

**Fig 1:**
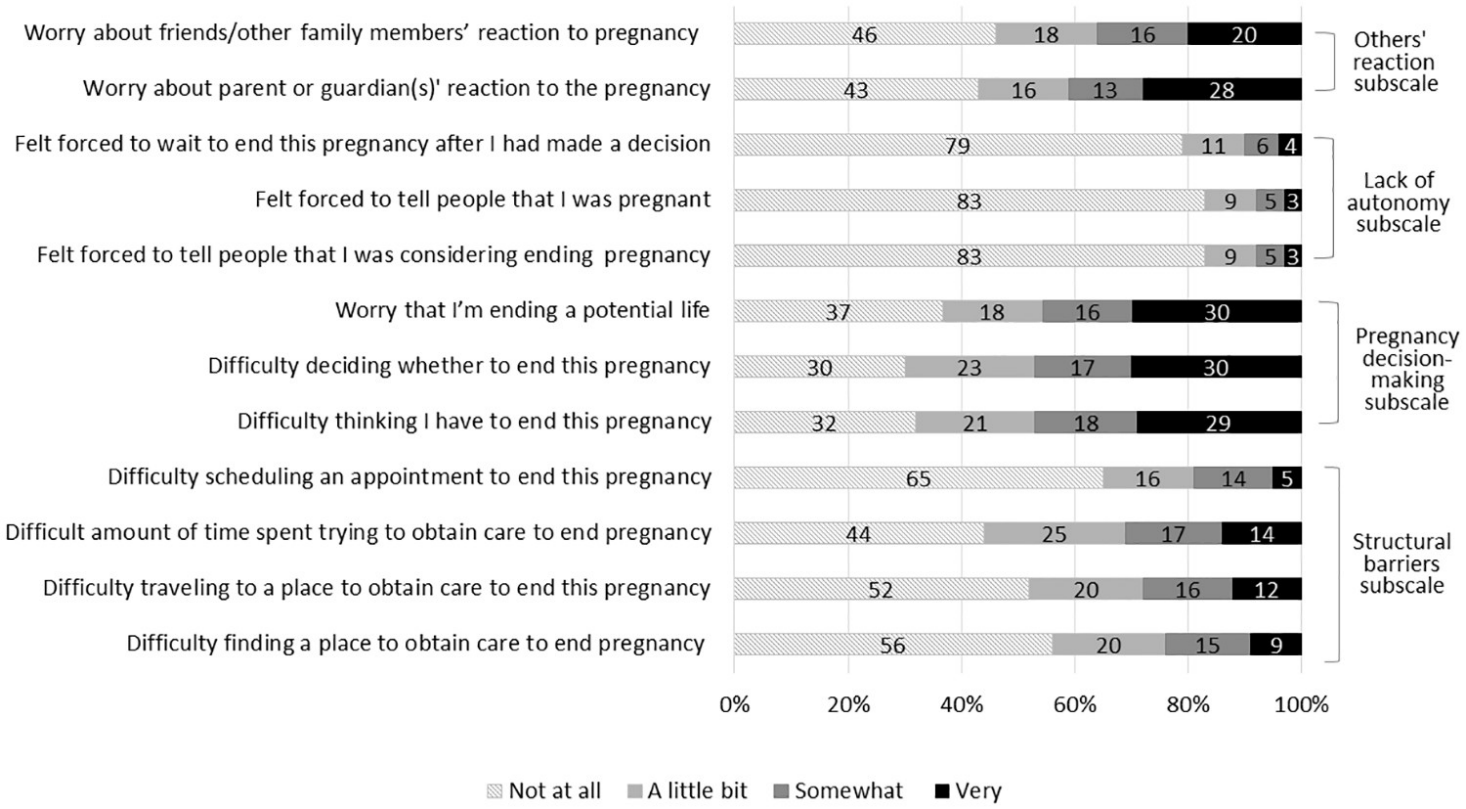
Distribution of responses to items comprising the Psychosocial Burden Seeking Abortion Scale (PB-SAS) and subscales.

Fig 1 presents the distribution of each item. While all items were skewed towards less psychosocial burden, they demonstrated adequate variability. The items that were most frequently endorsed included difficulty “thinking I have to end this pregnancy” (47% somewhat/very difficult) and “deciding whether to end this pregnancy” (47% somewhat/very difficult), whereas the items under Factor 3 were the least likely to be endorsed: “I felt forced to tell people that I was pregnant” (8% somewhat/very much), “I felt forced to tell people that I was considering ending this pregnancy” (10% somewhat/very much), and “I felt forced to wait to end this pregnancy after I had made a decision” (8% somewhat/very much).

Correlations of the rotated common factors (Table 3) indicate that each factor is somewhat weakly correlated with each other (≤ 0.41) yet strongly associated with the full scale (≥ 0.60), suggesting that each factor is measuring independent yet related dimensions of *psychosocial burden*. We scored the full scale as a continuous measure by calculating the mean of all items (sum of all answered items divided by the total number of items answered) and similarly scored each sub-scale. In scoring the full scale, we only scored responses for people who had responded to at least 8 out of the 12 total items, and who had completed over half of the items for each subscale (3 out of 4 items for Factor 1, 2 out of 3 items for Factors 2 and 3, and both items for Factor 4).

**Table 3:** Correlation matrix of common factors.

Factors	Factor 1	Factor 2	Factor 3	Factor 4
Factor 1. Structural challenges	1.00			
Factor 2. Pregnancy decision-making	0.31	1.00		
Factor 3. Lack of autonomy	0.41	0.30	1.00	
Factor 4. Others’ reactions to the pregnancy	0.31	0.37	0.37	1.00
Full scale*	0.74	0.72	0.62	0.64

*Based on pairwise correlations.

CFAs exhibited satisfactory fit for the model with four correlated lower-order factors (CFI = .982, RMSEA = .036) and the model with a single higher-order factor representing the correlations among the four lower-order factors (CFI = .981, RMSEA = .046). The BIC comparison strongly favored the higher-order factor model (BIC = 22627.856) over the correlated lower-order factors model (BIC = 22634.400; difference = 6.544), suggesting that, in general, researchers should use the full scale in future studies.

In adjusted analyses, the overall scale was significantly associated with perceived stress (β = 1.71, 95% Confidence Interval 1.30, 2.13), generalized anxiety (β = 3.52, 95% CI 2.74 to 4.31), and depressive symptoms (β = 0.93, 95% CI 0.67 to 1.19, Table 4). Each subscale was also significantly associated with each mental health outcome (Table 5). By running each model with and without the full scale, we estimated Cohen’s f^2^ which were 0.10, 0.11, and 0.08, respectively, representing medium effect sizes [39]. As an alternative measure of effect sizes, we present the unadjusted pairwise correlations between the full scale, subscales and mental health measures (S2 Table) as well as the pairwise correlations among each individual scale item (S3 Table) separately.

**Table 4:** Association between full scale and symptoms of perceived stress, generalized anxiety, and depression, according to linear multivariable regression analyses.

	Perceived Stress (n = 729)		Generalized Anxiety (n = 725)		Depressive Symptoms (n = 722)	
	Coef. [95% CI]	*P*	Coef. [95% CI]	*P*	Coef. [95% CI]	*P*
Psychosocial Burden Scale (PB-SAS)	**1.71** [1.30,2.13]	< .01	**3.52** [2.74,4.31]	< .01	**0.93** [0.67,1.19]	< .01
Age group						
15–19 (Ref.)						
20–24	0.34 [-0.48,1.16]	0.42	0.61 [-0.95,2.17]	0.44	0.21 [-0.31,0.71]	0.43
25–29	0.32 [-0.51,1.15]	0.45	0.46 [-1.13,2.04]	0.57	0.08 [-0.44,0.60]	0.75
30–34	0.93 [-0.01,1.87]	0.05	0.38 [-1.40,2.16]	0.68	0.24 [-0.35,0.82]	0.43
35–45	0.24 [-0.77,1.27]	0.65	0.13 [-1.82,2.07]	0.90	0.23 [-0.41,0.86]	0.48
Race/ethnicity						
*White (non-Hispanic) (Ref*.*)*						
*Black (non-Hispanic)*	0.45 [-0.19,1.09]	0.17	0.20 [-1.02,1.43]	0.75	**0.62** [0.22,1.02]	< .01
*Hispanic/Latina*	0.28 [-0.37,0.93]	0.39	0.14 [-1.08,1.37]	0.82	**0.52** [0.12,0.93]	0.01
*Asian/Pacific Islander(non-Hispanic)*	-0.10 [-1.13,0.92]	0.85	0.99 [-0.95,2.94]	0.32	0.62 [-0.02,1.27]	0.06
*Other /Mixed race*	0.30 [-0.48,1.09]	0.45	-0.94 [-2.44,0.56]	0.22	0.08 [-0.41,0.58]	0.74
Marital status				0.75		
*Never married (Ref*.*)*						
*Married*	0.13 [-0.69,0.95]	0.76	0.01 [-1.55,1.56]	0.99	-0.02 [-0.53,0.49]	0.93
*Separated/Divorced/Widowed*	-0.67 [-1.47,0.12]	0.10	0.65 [-0.88,2.18]	0.41	-0.17 [-0.67,0.33]	0.51
Nulliparous	0.47 [-0.12,1.06]	0.12	**1.30** [0.16,2.44]	0.03	0.30 [-0.07,0.67]	0.11
In very committed intimate relationship with man involved in pregnancy	**-0.64** [-1.15,-0.14]	0.01	**1.02** [0.05,1.98]	0.04	-0.09 [-0.40,0.23]	0.59
Pre-pregnancy pregnancy preferences						
*Pregnancy not wanted (Ref*.*)*						
*Pregnancy mistimed*	-0.24 [-0.78,0.31]	0.39	-0.16 [-1.20,0.87]	0.76	-0.16 [-0.51,0.18]	0.36
*Pregnancy wanted*	-0.25 [-1.48,0.98]	0.69	0.58 [-1.76,2.92]	0.63	0.40 [-0.37,1.17]	0.31
*Not sure what wanted*	-0.09 [-0.72,0.53]	0.78	-0.11 [-1.30,1.08]	0.85	0.01 [-0.37,0.41]	0.93
Gestational age						
*< = 12 wks*. *(Ref*.*)*						
*13–19 wks*.	-0.12] [-0.83,0.59]	0.75	-1.30 [-2.66,0.06]	0.06	-0.27 [-0.72,0.18]	0.25
*> = 20 wks*.	0.34 [-0.43,1.11]	0.39	-0.00 [-1.47,1.47]	1.00	-0.17 [-0.65,0.31]	0.50
Abortion due to rape	0.57 [-1.13,2.28]	0.51	**3.57** [0.34,6.80]	0.03	0.90 [-0.16,1.96]	0.10
Abortion due to fetal anomaly	0.33 [-0.95,1.60]	0.61	1.06 [-1.36,3.48]	0.39	0.16 [-0.64,0.95]	0.70
Confidence could up with $2,000 if unexpected need arose within month						
*Not at all confident (Ref*.*)*						
*Only slightly confident*	**-0.60** [-1.18,-0.02]	< .05	-1.06 [-2.17,0.04]	0.06	-0.18 [-0.55,0.18]	0.32
*Somewhat confident*	**-0.86** [-1.51,-0.22]	0.01	-0.76 [-2.02,0.50]	0.24	-0.27 [-0.69,0.15]	0.21
*Very confident*	**-1.23** [-2.01,-0.45]	< .01	-1.07 [-2.57,0.43]	0.16	-0.11 [-0.61,0.38]	0.66
Any government assistance, last year	-0.26 [-0.77,0.26]	0.33	-0.14 [-1.13,0.85]	0.79	0.01 [-0.31,0.34]	0.94
**History of adversity and mental health problems**						
Anxiety or depression diagnosis	**1.10** [0.54,1.65]	< .01	**3.28** [2.24,4.33]	< .01	**1.01** [0.66,1.36]	< .01
Pre-pregnancy problem alcohol use	0.51 [0.00,1.02]	0.05	**1.55** [0.57,2.52]	< .01	**0.44** [0.12,0.76]	0.01
Pre-pregnancy illicit drug use	0.43 [-0.28,1.15]	0.23	0.32 [-1.04,1.69]	0.64	0.42 [-0.03,0.88]	0.07
History of childhood adversity	**0.65** [0.14,1.16]	0.01	**1.26** [0.30,2.23]	0.01	**0.40** [0.08,0.72]	0.01
*Site A (Ref*.*)*						
*B*	0.18 [-0.46,0.83]	0.58	-0.42 [-1.65,0.81]	0.50	-0.07 [-0.47,0.34]	0.75
*C*	0.37 [-0.34,1.07]	0.31	1.15 [-0.19,2.48]	0.09	0.04 [-0.40,0.48]	0.87
*D*	0.31 [-0.59,1.22]	0.50	0.72 [-1.00,2.43]	0.41	-0.04 [-0.61,0.52]	0.88
Cohen’s f^2^	*0*.*10*		*0*.*11*		*0*.*08*	

Ref. = Reference group, Coef = Beta coefficient, CI: Confidence Interval; **Bold** items significant at p < .05. Cohen’s f^2^ is based on Fisher’s z transformation and relates to the specific predictive contribution of each subscale.

**Table 5:** Association between subscales and symptoms of perceived stress, generalized anxiety, and depression, according to linear multivariable regression analyses.

	Beta Coefficient [95% Confidence Intervals]	p value	Cohen’s f^2^
	**Perceived Stress (N = 729)**		
Factor 1. Structural challenges	0.82[0.51,1.29]	p < .001	0.04
Factor 2. Pregnancy decision	0.79[0.56,1.02]	p < .001	0.06
Factor 3. Lack of autonomy	0.94[0.56,1.32]	p < .001	0.03
Factor 4. Others’ reactions to the pregnancy	0.43[0.21,0.65]	p < .001	0.02
	**Generalized Anxiety (N = 725)**		
Factor 1. Structural challenges	1.74[1.47,2.32]	p < .001	0.05
Factor 2. Pregnancy decision	1.78[1.33,2.21]	p < .001	0.10
Factor 3. Lack of autonomy	1.58[0.84,2.31]	p < .001	0.03
Factor 4. Others’ reactions to the pregnancy	0.82[0.39,1.25]	p < .001	0.03
	**Depressive Symptoms (N = 725)**		
Factor 1. Structural challenges	0.44[0.25,0.63]	p < .001	0.04
Factor 2. Pregnancy decision	0.49[0.35,0.64]	p < .001	0.06
Factor 3. Lack of autonomy	0.50[0.26,0.74]	p < .001	0.02
Factor 4. Others’ reactions to the pregnancy	0.15[0.01,0.29]	0.04	0.01

Note all models use multiple imputation methods for model covariates and adjust for age, race/ethnicity, marital status, parity, relationship status, pregnancy intentions, gestational age, reason for abortion, socioeconomic status, and history of childhood adversity and mental health problems; Cohen’s f^2^ is based on Fisher’s z transformation and relates to the specific predictive contribution of each subscale.

## Discussion

We offer a new practical 12-item scale that can be used by providers and researchers to empirically measure the psychosocial burden experienced by people seeking abortion. We hypothesized that psychosocial burden is a multi-faceted construct, encompassing both individual and structural-level factors. Indeed, we identified four key domains that comprise the psychosocial burden felt by people wanting abortion. These include logistical barriers accessing abortion care, the pregnancy decision, lack of autonomy, and concern about other people’s reactions to the pregnancy. Preliminary construct validity was established through EFA. Each subscale had strong correlations with the overall scale and weaker correlations with the other subscales, indicating that they are measuring distinct, yet related domains and can be used as independent measures. With alpha levels well above the recommended score of 0.70 [30], this novel measure has high internal consistency reliability, demonstrating that the items function well together to measure people’s experience with psychosocial burden during the process of obtaining an abortion. We recommend using the full scale as a continuous measure to assess overall psychosocial burden. However, for researchers interested in a specific subset of psychosocial burden we believe the sub-scales can also be used as independent measures. We recommend using the scale among anyone seeking abortion, including people from other countries, although we also suggest further scale validation among diverse populations.

Predictive validity was established by the significant associations between the scale and three distinct mental health outcomes: stress, anxiety, and depression. Psychosocial burden scores skewed low, in particular for the items related to lack of autonomy which included feeling forced to tell people about the abortion or pregnancy and to wait to have the abortion after having made the decision. However, while threats to autonomy were not commonly experienced, their significant association with each mental health outcome highlights the importance of preserving people’s autonomous decision-making. The addition of the overall scale and each subscale as predictors of mental health, demonstrated medium effect sizes. The full scale had the largest strength of association with anxiety, followed by stress and depressive symptoms. Among subscales, the largest strength of association was between pregnancy decision-making and symptoms of anxiety.

This study is among the first we are aware of to quantitatively demonstrate that the logistical barriers accessing abortion care are associated with mental health symptoms. The first factor, structural challenges, was composed of four items related to the barriers accessing abortion care—travel, finding a place, the amount of time accessing care, and scheduling an appointment. This factor explained the largest proportion of the variability in the overall scale. Its significant relationship with mental health symptoms indicates that laws that reduce the number of available clinics [40], increase the distances traveled [41], and make abortion less accessible [42] may be harmful to people’s mental health. The most recent Supreme Court decision in June Medical Services vs Russo [43] reaffirmed that laws that place an “undue burden” on people’s right to an abortion are unconstitutional. Yet burdensome restrictions remain widespread, despite the lack of evidence demonstrating whether these restrictions benefit people seeking abortion, and as shown in this study, may contribute to more elevated levels of psychological distress. Efforts to de-medicalize abortion by making medication abortion available through telemedicine or over-the-counter without a prescription would likely decrease people’s psychosocial burden accessing abortion [44–46], and should be explored through further study.

When we examined factor two, pregnancy decision-making, which included difficulty thinking they have to end the pregnancy, difficulty deciding whether to end the pregnancy, and feeling worried that they are ending a potential life, over half of the sample endorsed these statements. This finding is strikingly similar to other studies of people seeking abortion. For example, in the Turnaway Study, just over half (56%) of people seeking an abortion described the decision as somewhat or very difficult. Importantly, the vast majority (95%) of Turnaway Study participants also reported that abortion was the right decision for them, both one week after they sought care and over five years [47]. Difficulty making a decision is expected when a decision involves weighing complex risks and benefits and is sensitive to personal preferences and values; prior research indicates that many medical decisions ranging from whether to undergo invasive prenatal testing to how aggressively to treat cancer involve some level of conflict around the decision [48]. Decision-making about pregnancy requires people to assess their existing financial, spiritual, emotional or social context and resources and weigh multiple risks and benefits as they choose the option that is best for them. A recent cross-sectional study of pregnant people found that people choosing abortion and people choosing to continue the pregnancy reported similar levels of decision conflict [49]. Thus, we find that just as people may experience some difficulty making other healthcare decisions, abortion is similar.

Importantly, while we found that people seeking abortion commonly experience some difficulty around the decision, we also found that lack of autonomy emerged as a key psychosocial burden domain which was significantly associated with symptoms of stress, anxiety and depression. Factor three or lack of autonomy, included feeling forced to disclose the pregnancy decision and feeling forced to wait to have the abortion after having made the decision, as is required in states with mandated waiting period laws. A prospective study of people presenting for abortion in Utah which has a mandated 72-hour waiting period law, found that forcing people to wait resulted in unwanted disclosure about the abortion [50]. About 6% of people had to disclose that they were considering abortion in order to make the logistical arrangements for the abortion information visit [50]. In the current study, about one in ten people endorsed the statement that they felt forced to disclose they were considering abortion and a similar proportion felt forced to wait to have the abortion after they had already made a decision. Research from Illinois found that enforcement of a parental notification law resulted in a slight increase in the proportion of young people who felt forced to have an abortion [51]. Our findings suggest that mandatory waiting periods, parental involvement requirements, and other legal restrictions that reduce people’s autonomy around the pregnancy decision are likely to increase people’s psychosocial burden and their risk for experiencing mental health symptoms.

Concerns about other people’s reactions to the pregnancy emerged as the fourth psychosocial burden domain among people seeking abortion. People who reported concerns about the reactions from parents, friends and other people in their lives are likely perceiving some stigma around the pregnancy decision. This finding echoes other work which has demonstrated that most people seeking abortion perceive and internalize stigma around the abortion and that this contributes to higher levels of psychological distress up to five years after abortion-seeking [9,52]. Similarly, other research has found that when young people involve an unsupportive parent, they are more likely to anticipate poor coping after the abortion [53].

### Limitations and strengths

This study has a number of limitations that should be considered. First, because our sample is limited to people accessing clinic-based abortion care, it likely overrepresents the experiences of people with the economic and emotional resources to overcome barriers to care. Thus, levels of psychosocial burden might be higher among people who consider abortion but do not present for abortion care. On the other hand, our sample may also overrepresent people experiencing travel burden and delay accessing care since participants were more likely to travel more than 25 miles and to present for care later in pregnancy when compared to people seeking abortion nationally [54]. Furthermore, our cross-sectional design does not allow us to make causal inferences or to assess the potential longer-term effects of psychosocial burden on people‘s psychological well-being. Nonetheless, our large sample size, representing a spectrum of ages, racial/ethnic groups, gestational age categories, pregnancy intentions, and people living in as many as 20 different states within the U.S., allowed us to examine a broad range of abortion-seeking experiences. Our sample was mostly similar in terms of age, race/ethnicity and marital status when compared to a national sample of people presenting for care [54]. Additional study strengths include our use of widely accepted mental health measures that have been used extensively and validated in the United States and elsewhere, as well as the inclusion of a broad range of abortion-related topics into one measure. By examining a broad range of domains we are able to describe multiple components of the abortion seeking experience that contribute to people’s psychological well-being.

### Conclusions

We provide a validated tool to measure the psychosocial burden experienced by people seeking abortion in the United States. We find four distinct dimensions of psychosocial burden, each of which are associated with people’s psychological well-being. Any legal restrictions that increase travel burden, unwanted abortion disclosure, and force people to wait are likely affecting people’s psychological well-being. In fact, the same restrictions that claim to protect people from mental health harm, may increase people’s psychosocial burden and contribute to adverse psychological outcomes at the time of seeking abortion.

## Supporting information

S1 Table Psychosocial Burden among people Seeking Abortion Scale (PB-SAS). (PDF)

S2 Table Pairwise correlation matrix of scale, subscales and mental health measures. (PDF)

S3 Table Pairwise correlation matrix of individual scale items. (PDF)

## References

[1] Biggs MA, Upadhyay UD, McCulloch CE, Foster DG. Women’s Mental Health and Well-being 5 Years After Receiving or Being Denied an Abortion: A Prospective, Longitudinal Cohort Study. JAMA Psychiatry 2017;74:169–78. 10.1001/jamapsychiatry.2016.3478 27973641

[2] Major B, Cozzarelli C, Cooper ML, Zubek J, Richards C, Wilhite M, et al Psychological Responses of Women After First-Trimester Abortion. Arch Gen Psychiatry 2000;57:777–84. 10.1001/archpsyc.57.8.777 10920466

[3] Harris LF, Roberts SCM, Biggs MA, Rocca CH, Foster DG. Perceived stress and emotional social support among women who are denied or receive abortions in the United States: a prospective cohort study. BMC Womens Health 2014;14:76 10.1186/1472-6874-14-76 24946971 PMC4080695

[4] National Academies of Sciences E. The Safety and Quality of Abortion Care in the United States. 2018. 10.17226/24950. 29897702

[5] Munk-Olsen T, Laursen TM, Pedersen CB, Lidegaard Ø, Mortensen PB. Induced first-trimester abortion and risk of mental disorder. N Engl J Med 2011;364:332–9. 10.1056/NEJMoa0905882 21268725

[6] Chae S, Desai S, Crowell M, Sedgh G. Reasons why women have induced abortions: a synthesis of findings from 14 countries. Contraception 2017;96:233–41. 10.1016/j.contraception.2017.06.014 28694165 PMC5957082

[7] Biggs MA, Gould H, Foster DG. Understanding why women seek abortions in the US. BMC Womens Health 2013;13:29 10.1186/1472-6874-13-29 23829590 PMC3729671

[8] Rocca CH, Samari G, Foster DG, Gould H, Kimport K. Emotions and decision rightness over five years following an abortion: An examination of decision difficulty and abortion stigma. Soc Sci Med 2020;248:112704 10.1016/j.socscimed.2019.112704 31941577

[9] Biggs MA, Brown K, Foster DG. Perceived abortion stigma and psychological well-being over five years after receiving or being denied an abortion. PloS One 2020;15:e0226417 10.1371/journal.pone.0226417 31995559 PMC6988908

[10] Guttmacher Institute. Counseling and Waiting Periods for Abortion. 2020. (accessed May 19, 2020). https://www.guttmacher.org/state-policy/explore/counseling-and-waiting-periods-abortion

[11] Texas Department of State Health Services and Texas Health and Human Services Commission [Internet]. A woman’s right to know. Revised December 2016. (accessed Nov 2020). https://hhs.texas.gov/sites/default/files/documents/services/health/women-children/womans-right-to-know.pdf

[12] Steinberg JR, Laursen TM, Adler NE, Gasse C, Agerbo E, Munk-Olsen T. The association between first abortion and first-time non-fatal suicide attempt: a longitudinal cohort study of Danish population registries. Lancet Psychiatry 2019;6:1031–8. 10.1016/S2215-0366(19)30400-6 31757590 PMC7437954

[13] Biggs MA, Gould H, Barar RE, Foster DG. Five-Year Suicidal Ideation Trajectories Among Women Receiving or Being Denied an Abortion. Am J Psychiatry 2018;175:845–52. 10.1176/appi.ajp.2018.18010091 29792049

[14] Biggs MA, Rowland B, McCulloch CE, Foster DG. Does abortion increase women’s risk for post-traumatic stress? Findings from a prospective longitudinal cohort study. BMJ Open 2016;6:e009698 10.1136/bmjopen-2015-009698 26832431 PMC4746441

[15] Major B, Appelbaum M, Beckman L, Dutton MA, Russo NF, West C. Abortion and mental health: Evaluating the evidence. Am Psychol 2009;64:863–90. 10.1037/a0017497 19968372

[16] Foster DG, Kimport K. Who seeks abortions at or after 20 weeks? Perspect Sex Reprod Health 2013;45:210–8. 10.1363/4521013 24188634

[17] Janiak E, Kawachi I, Goldberg A, Gottlieb B. Abortion barriers and perceptions of gestational age among women seeking abortion care in the latter half of the second trimester. Contraception 2014;89:322–7. 10.1016/j.contraception.2013.11.009 24332434

[18] Upadhyay UD, Weitz TA, Jones RK, Barar RE, Foster DG. Denial of abortion because of provider gestational age limits in the United States. Am J Public Health 2014;104:1687–94. 10.2105/AJPH.2013.301378 23948000 PMC4151926

[19] Jerman J, Frohwirth L, Kavanaugh ML, Blades N. Barriers to Abortion Care and Their Consequences For Patients Traveling for Services: Qualitative Findings from Two States. Perspect Sex Reprod Health 2017;49:95–102. 10.1363/psrh.12024 28394463 PMC5953191

[20] Coleman-Minahan K, Stevenson AJ, Obront E, Hays S. Young Women’s Experiences Obtaining Judicial Bypass for Abortion in Texas. J Adolesc Health Off Publ Soc Adolesc Med 2019;64:20–5. 10.1016/j.jadohealth.2018.07.017 30197199 PMC7274206

[21] Lazarus RS, Folkman S. Stress, appraisal, and coping. Springer publishing company; 1984.

[22] Hinkin TR, Tracey JB, Enz CA. Scale construction: Developing reliable and valid measurement instruments. J Hosp Tour Res 1997;21:100–120.

[23] Alderdice F, Lynn F, Lobel M. A review and psychometric evaluation of pregnancy-specific stress measures. J Psychosom Obstet Gynecol 2012;33:62–77. 10.3109/0167482X.2012.673040 22554138

[24] Yali AM, Lobel M. Coping and distress in pregnancy: an investigation of medically high risk women. J Psychosom Obstet Gynecol 1999;20:39–52. 10.3109/01674829909075575 10212886

[25] Cohen S, Kamarck T, Mermelstein R. A global measure of perceived stress. J Health Soc Behav 1983:385–396. 6668417

[26] Kanner AD, Coyne JC, Schaefer C, Lazarus RS. Comparison of two modes of stress measurement: Daily hassles and uplifts versus major life events. J Behav Med 1981;4:1–39. 10.1007/BF00844845 7288876

[27] Nunnally JC, Bernstein IH. Psychological theory 1994.

[28] Cureton EE, D’Agostino RB. Factor analysis: An applied approach. Psychology press; 2013.

[29] Child D. The Essentials of Factor Analysis, 3rd Edition Continuum International Publishing New York, NY 2006.

[30] Cortina JM. What is Coefficient alpha? An Examination of Theory and Applications. J Appl Psychol. 1993;98–104.

[31] Bentler PM, Bonett DG. Significance tests and goodness of fit in the analysis of covariance structures. Psychol Bull 1980;88:588–606. 10.1037/0033-2909.88.3.588.

[32] Browne MW, Cudek R. Alternative Ways of Assessing Model Fit—Michael W. Browne, Robert Cudeck, 1992. Test. Struct. Equ. Models, Newbury Park, CA: Sage publications; n.d., p. 136–62.

[33] Hu L, Bentler PM. Cutoff criteria for fit indexes in covariance structure analysis: Conventional criteria versus new alternatives. Struct Equ Model Multidiscip J 1999;6:1–55. 10.1080/10705519909540118.

[34] Raftery AE. Bayesian Model Selection in Social Research. Sociol Methodol 1995;25:111–63. 10.2307/271063.

[35] Hippel PTV. Regression with Missing Ys: An Improved Strategy for Analyzing Multiply Imputed Data. Sociol Methodol 2007;37:83–117. 10.1111/j.1467-9531.2007.00180.x.

[36] Spitzer RL, Kroenke K, Williams JBW, Löwe B. A brief measure for assessing generalized anxiety disorder: the GAD-7. Arch Intern Med 2006;166:1092–7. 10.1001/archinte.166.10.1092 16717171

[37] Kroenke K, Spitzer RL, Williams JBW. The Patient Health Questionnaire-2: validity of a two-item depression screener. Med Care 2003;41:1284–92. 10.1097/01.MLR.0000093487.78664.3C 14583691

[38] Finkelhor D, Shattuck A, Turner H, Hamby S. Improving the adverse childhood experiences study scale. JAMA Pediatr 2013;167:70–75. 10.1001/jamapediatrics.2013.420 23403625

[39] Cohen J. Statistical power analysis for the behavioral sciences. Academic press; 2013.

[40] Gerdts C, Fuentes L, Grossman D, White K, Keefe-Oates B, Baum SE, et al Impact of Clinic Closures on Women Obtaining Abortion Services After Implementation of a Restrictive Law in Texas. Am J Public Health 2016;106:857–64. 10.2105/AJPH.2016.303134 26985603 PMC4985084

[41] Kavanaugh ML, Jerman J, Frohwirth L. “It’s not something you talk about really”: information barriers encountered by women who travel long distances for abortion care. Contraception 2019;100:79–84. 10.1016/j.contraception.2019.03.048 30980828 PMC6589392

[42] Roberts SCM, Fuentes L, Kriz R, Williams V, Upadhyay UD. Implications for women of Louisiana’s law requiring abortion providers to have hospital admitting privileges. Contraception 2015;91:368–72. 10.1016/j.contraception.2015.02.001 25744615

[43] June Medical Services v. Russo, No. 18–1323. 2020.

[44] Kapp N, Grossman D, Jackson E, Castleman L, Brahmi D. A research agenda for moving early medical pregnancy termination over the counter. BJOG Int J Obstet Gynaecol 2017;124:1646–52. 10.1111/1471-0528.14646 28317327 PMC5637897

[45] Biggs MA, Ralph L, Raifman S, Foster DG, Grossman D. Support for and interest in alternative models of medication abortion provision among a national probability sample of U.S. women. Contraception 2019;99:118–24. 10.1016/j.contraception.2018.10.007 30448203

[46] Thompson T-A, Sonalkar S, Butler JL, Grossman D. Telemedicine for Family Planning: A Scoping Review. Obstet Gynecol Clin North Am 2020;47:287–316. 10.1016/j.ogc.2020.02.004 32451019 PMC10093687

[47] Rocca CH, Kimport K, Gould H, Foster DG. Women’s emotions one week after receiving or being denied an abortion in the United States. Perspect Sex Reprod Health 2013;45:122–31. 10.1363/4512213 24020773

[48] Ralph LJ, Foster DG, Kimport K, Turok D, Roberts SCM. Measuring decisional certainty among women seeking abortion. Contraception 2017;95:269–78. 10.1016/j.contraception.2016.09.008 27745910

[49] Rowland B, Rocca C, Ralph L. Certainty and Intention in Pregnancy Decision-making: an exploratory study. Contraception 2020;101:357–8. 10.1016/j.contraception.2020.03.017. PMC8186868 33189708

[50] Roberts SCM, Turok DK, Belusa E, Combellick S, Upadhyay UD. Utah’s 72-Hour Waiting Period for Abortion: Experiences Among a Clinic-Based Sample of Women. Perspect Sex Reprod Health 2016;48:179–87. 10.1363/48e8216 27010515

[51] Ralph LJ, King E, Belusa E, Foster DG, Brindis CD, Biggs MA. The Impact of a Parental Notification Requirement on Illinois Minors’ Access to and Decision-Making Around Abortion. J Adolesc Health Off Publ Soc Adolesc Med 2018;62:281–7. 10.1016/j.jadohealth.2017.09.031. 29248391

[52] Shellenberg KM, Tsui AO. Correlates of perceived and internalized stigma among abortion patients in the USA: an exploration by race and Hispanic ethnicity. Int J Gynaecol Obstet Off Organ Int Fed Gynaecol Obstet 2012;118 Suppl 2:S152–159. 10.1016/S0020-7292(12)60015-0 22920620

[53] Ralph L, Gould H, Baker A, Foster DG. The role of parents and partners in minors’ decisions to have an abortion and anticipated coping after abortion. J Adolesc Health Off Publ Soc Adolesc Med 2014;54:428–34. 10.1016/j.jadohealth.2013.09.021 24332398

[54] Jones RK, Jerman J. Population Group Abortion Rates and Lifetime Incidence of Abortion: United States, 2008–2014. Am J Public Health 2017;107:1904–9. 10.2105/AJPH.2017.304042 29048970 PMC5678377

