# Supplementary material for: Developing and validating the Psychosocial Burden among people Seeking Abortion Scale (PB-SAS)
Source: PLoS One. 2020 Dec 10;15(12):e0242463. doi: 10.1371/journal.pone.0242463 (PMC7728247; doi:10.1371/journal.pone.0242463)
Supplement: S1 Table — (PDF) [file pone.0242463.s001.pdf]

S1 Table. Psychosocial Burden Seeking Abortion Scale (PB-SAS)

The following questions ask you about your experiences seeking care to end this pregnancy. This may include using pills (medication abortion), having an aspiration or surgical abortion procedure, a D&E (dilation and evacuation), or an induced miscarriage or termination.

In thinking about the time since you discovered you were pregnant, please mark the choice that best describes…
If the statement does not feel true to you, then mark "not at all difficult" or "not at all"

**How difficult the following have been for you.**	**Not at all difficult**	**A little bit difficult**	**Somewhat difficult**	**Very difficult**	**Subscale**
Finding a place to obtain care to end this pregnancy	0	1	2	3	Structural Barriers
Scheduling an appointment to end this pregnancy	0	1	2	3	Structural Barriers
Traveling to a place to obtain care to end this pregnancy	0	1	2	3	Structural Barriers
The amount of time I have spent trying to obtain care to end this pregnancy	0	1	2	3	Structural Barriers
Deciding whether to end this pregnancy	0	1	2	3	Pregnancy decision-making
Thinking I have to end this pregnancy	0	1	2	3	Pregnancy decision-making
**How you have felt, when thinking about the following:**	**Not at all**	**A little bit**	**Somewhat**	**Very much**	**Subscale**
I felt worried that I'm ending a potential life	0	1	2	3	Pregnancy decision-making
I felt forced to tell people that I was pregnant	0	1	2	3	Lack of autonomy
I felt forced to tell people that I was considering ending this pregnancy	0	1	2	3	Lack of autonomy
I felt forced to wait to end this pregnancy after I had made a decision	0	1	2	3	Lack of autonomy
I felt worried about my parent(s)' or guardian(s)' reaction to the pregnancy	0	1	2	3	Others' reactions
I felt worried about my friends' or other family members' reaction to the pregnancy	0	1	2	3	Others' reactions
Add score for each column	+	+	+	=	
Total Score	÷	(total items)=		Final score	

*Must answer at least 8 items to calculate full scale scores and more than half to calculate subscale scores

Cite as: Biggs, M.A., Neilands, T.B., Kaller, S., Wingo, E., Ralph, L.J. Developing and validating the Psychosocial Burden among people Seeking Abortion Scale (PB-SAS). 2020.
