# Supplementary material for: Developing and validating the Psychosocial Burden among people Seeking Abortion Scale (PB-SAS)
Source: PLoS One. 2020 Dec 10;15(12):e0242463. doi: 10.1371/journal.pone.0242463 (PMC7728247; doi:10.1371/journal.pone.0242463)
Supplement: S2 Table — (PDF) [file pone.0242463.s002.pdf]

S2 Table. Pairwise correlation matrix of scale, subscales and mental health measures

**Factors**	**Stress**	**Anxiety**	**Depression**
Factor 1. Structural challenges	0.29	0.29	0.21
Factor 2. Pregnancy decision-making	0.30	0.33	0.27
Factor 3. Lack of autonomy	0.28	0.24	0.23
Factor 4. Others' reactions to the pregnancy	0.25	0.23	0.16
Full scale	0.40	0.41	0.32

*All values are statistically significant at p<.001.
