# Supplementary material for: Developing and validating the Psychosocial Burden among people Seeking Abortion Scale (PB-SAS)
Source: PLoS One. 2020 Dec 10;15(12):e0242463. doi: 10.1371/journal.pone.0242463 (PMC7728247; doi:10.1371/journal.pone.0242463)
Supplement: S3 Table — (PDF) [file pone.0242463.s003.pdf]

S3 Table. Pairwise correlation matrix of individual scale items

Items	1	2	3	4	5	6	7	8	9	10	11	12
1. Finding a place to obtain care to end this pregnancy	1.00											
2. Scheduling an appointment to end this pregnancy	0.59	1.00										
3. Traveling to a place to obtain care to end this pregnancy	0.61	0.57	1.00									
4. The amount of time I have spent trying to obtain care to end this pregnancy	0.59	0.54	0.60	1.00								
5. Deciding whether to end this pregnancy	0.15	0.22	0.16	0.21	1.00							
6. Thinking I have to end this pregnancy	0.18	0.24	0.18	0.22	0.82	1.00						
7. I felt worried that I'm ending a potential life	0.18	0.22	0.21	0.24	0.58	0.59	1.00					
8. I felt forced to tell people that I was pregnant	0.24	0.25	0.21	0.28	0.14	0.14	0.19	1.00				
9. I felt forced to tell people that I was considering ending this pregnancy	0.20	0.23	0.22	0.25	0.21	0.22	0.23	0.69	1.00			
10. I felt forced to wait to end this pregnancy after I had made a decision	0.26	0.31	0.28	0.28	0.20	0.22	0.24	0.54	0.57	1.00		
11. I felt worried about my parent(s)' or guardian(s)' reaction to the pregnancy	0.17	0.20	0.21	0.23	0.22	0.23	0.33	0.25	0.20	0.22	1.00	
12. I felt worried about my friends' or other family members' reaction to the pregnancy	0.16	0.19	0.18	0.23	0.24	0.25	0.34	0.30	0.26	0.26	0.74	1.00

*All values are statistically significant at p<.001.
